# Autonomous Exploration in Unknown Indoor 2D Environments Using Harmonic Fields and Monte Carlo Integration

**DOI:** 10.3390/s25164894

**Published:** 2025-08-08

**Authors:** Dimitrios Kotsinis, George C. Karras, Charalampos P. Bechlioulis

**Affiliations:** 1 Division of Systems and Automatic Control, Department of Electrical and Computer Engineering, University of Patras, Rio, 26504 Patras, Greece; 2 Athena Research Center, Robotics Institute, Artemidos 6 & Epidavrou, 15125 Maroussi, Greece; gkarras@uth.gr; 3 Department of Informatics and Telecommunications, University of Thessaly, 35100 Lamia, Greece

**Keywords:** autonomous exploration, Poisson equation, Monte Carlo integration (MCI), walking on sphere (WoS), hybrid visibility graph

## Abstract

Efficient autonomous exploration in unknown obstacle cluttered environments with interior obstacles remains a challenging task for mobile robots. In this work, we present a novel exploration process for a non-holonomic agent exploring 2D spaces using onboard LiDAR sensing. The proposed method generates velocity commands based on the calculation of the solution of an elliptic Partial Differential Equation with Dirichlet boundary conditions. While solving Laplace’s equation yields collision-free motion towards the free space boundary, the agent may become trapped in regions distant from free frontiers, where the potential field becomes almost flat, and consequently the agent’s velocity nullifies as the gradient vanishes. To address this, we solve a Poisson equation, introducing a source point on the free explored boundary which is located at the closest point from the agent and attracts it towards unexplored regions. The source values are determined by an exponential function based on the shortest path of a Hybrid Visibility Graph, a graph that models the explored space and connects obstacle regions via minimum-length edges. The computational process we apply is based on the Walking on Sphere algorithm, a method that employs Brownian motion and Monte Carlo Integration and ensures efficient calculation. We validate the approach using a real-world platform; an AmigoBot equipped with a LiDAR sensor, controlled via a ROS-MATLAB interface. Experimental results demonstrate that the proposed method provides smooth and deadlock-free navigation in complex, cluttered environments, highlighting its potential for robust autonomous exploration in unknown indoor spaces.

## 1. Introduction

Mobile autonomous exploration, also known as Active Simultaneous Localization and Mapping (A-SLAM) [1], in indoor environments, refers to the capability of robots to navigate and gather information in unknown environments with minimal or no human guidance. It is a challenging topic in the robotics field, as it seeks to address three interconnected challenges simultaneously: trajectory planning, localization, and mapping. Essentially, the primary objective for a mobile robot in such scenarios is to build a reliable map of an unknown region while navigating safely in it without any prior information.

This process relies on data collected from the robot’s onboard sensors, such as LiDAR [2], RGB-D cameras [3], and IMUs [4]. Such sensor data helps the robot to understand the surrounding environment and make intelligent decisions to move to the next state, expanding the explored space safely. The ability to autonomously map unknown areas is vital for numerous real-world applications, including domestic and service robotics, autonomous warehouse and factory operations, and also critical missions like search and rescue in disaster or hazardous areas without human responders to be in danger.

### 1.1. Related Works

Recent advances in robotics have led to the development of sophisticated algorithms that address the challenges of autonomous exploration. These exploration algorithms can be classified according to how they represent and exploit the exploration space to navigate and map unknown regions. One such category is the methods based on Reinforcement Learning (RL). In this method, the mobile robot learns an exploration policy by interacting with the environment and maximizing a reward function that encourages efficient coverage of the map and goal-directed behavior in unknown spaces. In the research community, the RL method is state-of-the-art, and a great number of works have been presented. In [5], the authors propose a Cumulative Curriculum Reinforcement Learning (CCRL) framework to enhance Deep RL-based autonomous exploration by introducing a size-adaptive map representation and accelerating training through a custom lightweight grid-based simulator. The work in [6] presents a Deep RL-based exploration architecture that predicts long-term visiting sequences for unexplored sub-regions and integrates Next Best of View (NBV) selection. And finally, the authors in [7] develop a Graph-based Spatiotemporal Neural Network (G-STNN) combined with Deep RL to guide autonomous robot exploration, using previous trajectories and exploration space boundaries for enhancing the decision-making. Similar approaches have been presented in [8,9,10,11].

Another class of algorithms is based on sampling methods, where the robot randomly samples points within the explored space and selects the next best goal to expand it. They usually use motion graph planners [12] to navigate through an unknown area to the next state following utility-based criteria. For example, in [13], the authors propose a Dynamic Exploration Planner (DEP) that uses incremental sampling and Probabilistic Roadmaps to efficiently and safely explore dynamic environments by optimizing paths based on the NBV point. In [14], a Sampling-based Frontier-Block Detection (SFBD) method is presented that improves RRT-based exploration by reducing redundant sampling and costly nearest-point searches through a block-based structure for efficient collision avoidance and frontier detection. Finally, the work in [15] introduces an improved sampling-based exploration method that extends RRT until frontier endpoints are found and evaluates them using an enhanced utility function, leading to more efficient indoor exploration in 2D environments.

Another type of algorithms is based on the frontiers exploration. In these exploration algorithms, the mobile robot identifies and moves toward free boundaries to expand the explored region, exploiting the information from occupied and free explored boundaries. The paper of Yamauchi [16] was the first attempt that addressed this kind of problem, where the agent navigates to the free frontiers with A* algorithm. A class of frontier-based methods, on which our paper relies, is the Harmonic Potential Field (HPF). The HPF provides the agent with smooth navigation to unknown areas and easy implementation. These fields are typically derived from the solution of an elliptic Partial Differential Equation (PDE) with boundary conditions. We obtain their solution with numerical methods as the analytical solution is intractable [17]. A first attempt was presented in [18], which applies a HPF to explore an unknown area, solving with relaxation methods the Boundary Value Problem (BVP) with Dirichlet condition. That HPF leads the agent to the free frontiers (low values), avoiding collision with obstacles (high values). However, such kind of condition exhibits large flat areas of the potential, which result in the agent being stuck in regions which are far away from the frontier. Certain works proposed solutions of the above problem, such as [19], which applies potential rails that guide the robot to regions that are either unexplored or were visited a long time ago. The work in [20] develops a strategy to distort the potential field without loosing the qualities of the HPF, and the work in [21] enhances the BVP for integrated exploration by introducing active loop closure through dynamic barriers and a Voronoi-based local update window. Moreover, in the research community, there are projects which apply another boundary condition, the Neumann. The papers [22,23] solve the BVP using as a numerical method the Fast Multipole accelerated Boundary Element Method (FMBEM), which yields linear complexity in terms of computational effort and required memory. In [24], the authors use the panel method to create an HPF that leads the agent to the goal point. Finally, the paper [25] introduces a novel reactive, frontier-based exploration scheme that utilizes harmonic transformation to map a workspace onto a disk, ensuring safe navigation and enabling selective exploration of frontiers. Alternatively, Sawhney et al. [26] proposed another method to solve the BVP based on Brownsian Motion, known as the Walking on Sphere (WoS) Algorithm, which is a stochastic process applying Monte Carlo Integration (MCI) [27]. This method calculates the potential, and also its gradient, of a linear elliptic equation at any point in a connected region with different boundary value conditions, e.g., Dirichlet [26], Neumann [28], and Robin [29]. The aforementioned method is a grid-free algorithm and has been applied to geometrical problems in computer graphics exhibiting low computational cost.

### 1.2. Contributions

In this work, we address the problem of autonomous exploration for a mobile agent in an unknown indoor 2D environment that may contain interior obstacles. Our focus is on the map building problem and we assume the agent calculates its position via a certain localization process. We calculate velocity commands for navigating the agent by solving an elliptic PDE with Dirichlet condition over the explored space boundary. Most specifically, we solve a Poisson equation with a source point on the closest located free frontier. In this way, the agent escapes from potential flat regions created by the Dirichlet condition. The potential function associated with the source point is defined using a Hybrid Visibility Graph (HVG), which gives an estimate of the distance to the source point. The HVG involves the vertices and edges of the explored space boundary, as well as the minimum-length edges between the interior obstacles, enabling more accurate field estimation. Finally, we use the stochastic process WoS [26] for computational and memory efficiency. The key properties of our algorithm are (1) it is grid-free, i.e., it does not need to discretize the explored space to calculate the field and (2) we can calculate the gradient at the agent’s position without finding the potential field anywhere, thus reducing significantly the computational cost. We validated the approach using a real-world platform: an AmigoBot equipped with a LiDAR sensor, controlled via a ROS-MATLAB interface. Experimental results demonstrate that the proposed method provides smooth and deadlock-free navigation in complex, cluttered environments, highlighting its potential for robust autonomous exploration in unknown indoor spaces. In the results, we provide a comparative analysis of our method against other exploration approaches, aiming to present its advantages and disadvantages.

The rest of this paper is organized as follows: in Section 2, we formulate mathematically the exploration problem, in Section 3, we discuss the basic theory of elliptic PDEs with Dirichlet conditions and we present the WoS algorithm. In Section 4, we present the implementation of the proposed exploration process, and in Section 5, we describe the tools for realizing the real-world experiment. In Section 6, we present the results of the proposed exploration algorithm in three studies, and in Section 7, we discuss the results. Finally, we draw conclusions and future research directions.

### 1.3. Notations

#### 1.3.1. Mathematic Symbols

For any set $A\subset R^{n}$, we use ∂A to denote its boundary, int(A) to denote its interior, and |A| to denote its volume. We use $p^{A}$ to denote a probability density function on A and write $x∼p^{A}$ for a random point x∈A drawn from $p^{A}$. The uniform probability density function on A is U(A)=1/|A|. Moreover, we use B(x,r) to denote a ball contained in *n*-dimensional space $R^{n}$ with radius *r* centered at *x*. Additionally, $d\left(x,A\right)=min_{y\in \partial A}\left||y-x|\right|$ is denoted as the Euclidean distance from a point x∈A to the closest point on the ∂A and $\bar{x}=argmin_{y\in \partial A}||y-x||$ is denoted as the closet point. Furthermore, we define the ε-shell around ∂A as $\partial A^{\varepsilon }=\{x\in A:||x-\bar{x}\left||<\varepsilon \right\}$. Finally, we use Δ for the Laplace operator on $R^{n}$.

#### 1.3.2. Pseudocode Formation

For our exploration method, we introduce pseudocodes to clarify the WoS algorithm and the structure of the HVG. The notation for arrays and sets aligns with the MATLAB framework. Additionally, we denote a zero matrix as *zeros*(N,M) and a matrix of ones as *ones*(N,M), consistent with MATLAB properties.

## 2. Problem Formulation

In this section, we formulate our problem. First, we define the unknown workspace and the properties of the mobile agent, including its kinematic model and the onboard sensor model. Then, we discuss the properties of the explored region. Finally, we explain the data structure in which we store the unexplored region.

### 2.1. Workspace and Kinematics

We denote as *W* the unknown workspace, a compact and connected subset of $R^{2}$ and $W_{f}≜int\left(W\right)$ the free space. Let O=∂W consist of the mutually disjoint sets of the obstacle boundaries, $O_{i},i\in \left\{1,\ldots ,M\right\}$ as well as of the external boundary $O_{0}$, such that $O={⋃}_{i=0,\ldots ,M}O_{i}$. For this paper, we assume that the workspace *W* is time invariant and does not involve dynamic obstacles.

For simplicity, we use a differential mobile agent, which is a disk-shaped robot with radius ρ. The configuration space of the agent is defined as $W_{c}=W_{f}/\partial W^{\rho }$. The kinematic equation of a differential drive robot is given by:

(1) $$\left\{\begin{array}{c} {\dot{p}}_{x}=v_{l}\cdot cos\left(\theta \right)\\ {\dot{p}}_{y}=v_{l}\cdot sin\left(\theta \right)\\ \dot{\theta }=v_{a} \end{array}\right.$$

where $p={\left[p_{x},p_{y}\right]}^{T}$ is the position of the robot, the orientation of the agent is θ∈[−π,π], and $v_{l}$ and $v_{a}$ denote the linear and angular velocity of the agent with $v={\left[v_{l},v_{a}\right]}^{T}$. Notice that the agent relies on two motions to navigate in its free configuration space $W_{c}$; it rotates and moves forward. Moreover, we assume that the position and orientation of the agent are known through the exploration processes via a localization algorithm that assumes a known initial position $p_{0}$.

Additionally, we equip the agent with a range sensor, which gives the robot the capability to determine how close it is to the workspace boundary and explore unknown regions. The range sensor in each specific time step creates a sensing space $S\left(p;R_{max}\right)=\{p_{c}\in \partial W_{c}:||p-p_{c}\left|\right|\leq R_{max}\}$, where $R_{max}>0$ is the maximum range of the sensor. We assume that the minimum range $R_{min}$ of the sensor is rather small with $\rho >R_{min}$.

### 2.2. Explored Region

We define $P(t_{s},t_{f})$ as the path traversed by the agent during the time interval $\left[t_{s},t_{f}\right]$; for brevity, when $t_{s}=0$ and $t_{f}>0$, $P\left(t_{f}\right)\equiv P\left(0,t_{f}\right)$. Given a continuous path $P\left(t_{f}\right)\subset W_{c}$, we define the explored region of *W* as $E\left(P\left(t_{f}\right)\right)={⋃}_{p\in P}S\left(p;R_{max}\right)$. The boundary of the explored region at some time instance is denoted as $\partial E=\partial E_{f}\cup \partial E_{o}$, where $\partial E_{f}$ and $\partial E_{o}$ belong to the agent’s known free space $\partial E_{f}\subset W_{f}$ and workspace boundaries $\partial E_{o}\subseteq \partial W$, respectively. In general, each of $\partial E_{f}$ and $\partial E_{o}$ consists of zero or more disjoint line curves, that is, $\partial E_{f}={⋃}_{i\in I_{f}}\partial E_{fi}$ and $\partial E_{o}={⋃}_{i\in I_{o}}\partial E_{oi}$ with $I_{f}=\left\{1,2,\ldots ,N_{f}\right\}$ and $I_{o}=\left\{1,2,\ldots ,N_{o}\right\}$, indexing the distinct parts of the respective set.

One should note that any point $p\in \partial E_{f}$ that enters the agent’s sensing region becomes instantly part of int(E). In this way, as the agent approaches the free boundary, the interior of the explored space augments. In contrast, occupied boundary points will remain on the boundary, since if some point p∈∂W∩S, then p∈∂S [22]. So the problem that we try to solve is formulated as follows.

**Problem.**  *Design a control law u=f(p,θ,t,E) such that there exists a finite time instant T>0 for which E(P(t))=W,∀t≥T.*

### 2.3. Map Representation

The unknown workspace is discretized in a 2D grid, called an occupancy matrix [30]. We use this kind of matrix so that we can estimate the real form of the workspace. This grid is a 2D matrix consisting of cells $m_{i}$ centered at $c_{i}$, $i\in I_{G}$ with $I_{G}=\left\{1,2,\ldots ,N_{G}\right\}$. To associate each point p∈W with a cell, we define a map $m\left(p\right)=m_{i}$, where $\left|\right|c_{i}-p{\left|\right|}_{\infty }\leq \frac{m_{r}}{2}$ and $m_{r}$ denotes the grid resolution. We attach in each cell a binary occupancy value Pr(m(p)):W→[0,1], specifying whether the cell is free (Pr(m(p))→0), occupied (Pr(m(p))→1), or unknown (Pr(m(p))=0.5). In the exploration process, we use the binary occupancy map, which takes zero value for free and the unknown cells (Pr(m(p))=0) and true value for the occupied ones (Pr(m(p))=1). This structure is applied for simplicity and minimization of both memory and time complexity of the algorithm. Thus, we classify the points of the explored boundary, p∈∂E, as occupied or free and we also define the sets $\partial E_{o}=\left\{p\in \partial E:Pr\left(m\left(p\right)\right)=1\right\}$ and $\partial E_{f}=\left\{p\in \partial E:Pr\left(m\left(p\right)\right)=0\right\}$ or $\partial E_{f}=\partial E\setminus \partial E_{o}$. Finally, we initialize the values of the occupancy grid map with zero value.

**Remark 1.**  *
We apply the binary occupancy map for mapping because it is particularly simple and well suited for our proposed method. Unlike other methods that might benefit from detailed probabilistic representations, our approach does not necessitate intermediate probabilities of matrix cells. This choice contributes to the low memory and time complexity of our exploration method.
*

## 3. Solving PDEs with Monte Carlo Integration

### 3.1. Linear Elliptic Equation

The central idea behind the design of our exploration controller is to find a potential field φ(p):E→R by solving a Poisson equation, subject to boundary conditions that assign low potential to unexplored frontiers and high potential to boundaries corresponding to obstacles. The Poisson equation with pure Dirichlet boundary condition is:

(2) $$\begin{array}{cccc} \Delta \varphi (p) & =f(p), & \; & \;p\in E\\ \varphi (p) & =g(p), & \; & \;p\in \partial E \end{array}$$

Here, f(p):E→R is the source term and g(p):∂E→R.

Under the Dirichlet boundary condition, the values of the potential function at the boundaries are fixed, assigning the highest values to obstacles and the lowest to the unexplored boundary. Thus, this formulation generates a vector field that safely guides the agent towards the unexplored area. However, in complex environments, the agent may become trapped in regions distant from free frontiers, where the potential field becomes almost flat, and consequently the agent’s velocity nullifies as the gradient vanishes. To address this problem, a source point is introduced at the nearest free frontier relative to the agent’s position so that the agent moves away from low-gradient regions. The definition of the source function f(p) will be discussed later in the next section.

The solution of the Poisson equation at any point p∈E is based on the generalized mean value property of Laplace equation.

**Proposition** **1** (**Generalized Mean Value Property [31]**)**.** *If the boundary of the exploration space ∂E is sufficiently smooth, and the solution of (2) with the source term f(p) is continuous in E, then the solution of the Poisson Equation (2) attains the integral term:*

(3) $$\varphi \left(p\right)=\frac{1}{\left|\partial B(p)\right|}\int_{\partial B(p)}\varphi (y)dy+\int_{B(p)}f\left(y\right)G^{B(p)}\left(p,y\right)dy$$

*where B(p) is the largest ball contained in E with radius R centered at p, and $G^{B(p)}\left(p,y\right)$ is the Green’s function for the ball (see Appendix B).*

Notice that the above form attains a probabilistic interpretation, as the value φ(p) at the center of the sphere is the uniform average of the boundary values on the surface of the ball plus the average of the source term f(p) w.r.t. the Green function for the ball. Therefore, if the boundary values on the sphere are known, we may apply the MCI method (see Appendix A) to estimate the solution φ(p).

However, the solution of the Poisson equation is not sufficient. We have to calculate the gradient of the solution ∇φ(p) in order to determine the velocity commands of the controller. In [26], the authors provide the gradient of the solution based on Malliavin Calculus [32]:

(4) $$\nabla \varphi \left(p\right)=\frac{1}{\left|B(p)\right|}\int_{\partial B(p)}\varphi (y)\nu (y)dy+\int_{B(p)}f\left(y\right)\nabla G^{B(p)}\left(p,y\right)dy$$

where ν(y):=(y−p)/R is the outward unit normal at *y*.

### 3.2. The Walking on Sphere

In this subsection, we discuss the algorithm that we will use to find the solution of (2) and its gradient at a specific position in the explored region E, following the integral (4). According to [26], we can find the solution (3) following Kakutani’s principle.

**Definition 1** (**Kakutani’s principle [33]**)**.** *the solution value φ(p) at any point p∈E is equal to the expected value E[g(y)], where y∈∂E is the first boundary point reached by a random walk starting at p.*

**Figure 1:**
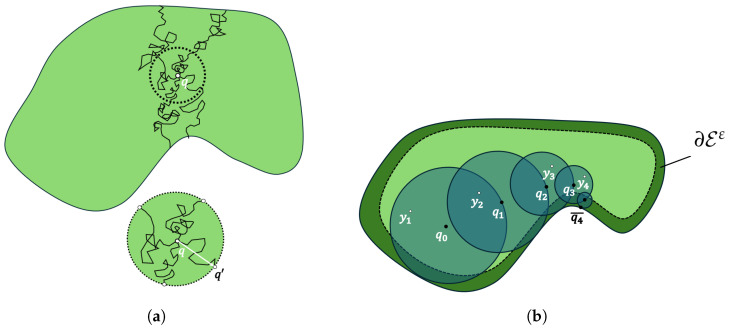
(**a**) Brownian motion process. (**b**) Walking on Sphere algorithm process finding the Poisson equation solution.

For simplicity, let us assume f(p)=0. Following the Kakutani’s principle or Brownian motion process, we can exploit the occupancy grid map by taking random steps from a starting cell $m(p_{0})$ until reaching ∂E and get the specific Dirichlet value. Nevertheless, this process has a lot of computational effort, as we decrease the resolution of the grid and the unexplored workspace is quite large. For that reason, we use a more efficient grid-free algorithm, which was proposed in [34], called *Walking on Sphere*. Essentially, this algorithm proposes taking the largest steps from the starting point $p_{0}$ over a sphere boundary centered at $p_{0}$ with radius the minimum distance to ∂E. This walk is equal to the walk occurring from Brownian motion, since the point that we take on the sphere is independent of which steps we take in the sphere, as we see in Figure 1a. Thus, the WoS algorithm provides less computational and memory complexity.

The WoS algorithm for finding the solution (3) of the Poisson equation relies on a recursive Monte Carlo single-sample estimator at point $p_{k}\in E$:

(5) $$\hat{\varphi }\left(p_{k}\right):=\left\{\begin{array}{cc} g(\bar{p_{k}}), & \mathrm{if}\;p_{k}\in \partial E^{\varepsilon }\\ \frac{1}{\left|\partial B(p_{k},r_{k})\right|}\frac{\hat{\varphi }\left(p_{k+1}\right)}{p^{\partial B(p_{k},r_{k})}\left(p_{k+1}\right)}+\frac{G^{B(p_{k},r_{k})}f\left(y_{k+1}\right)}{p^{B(p_{k},r_{k})}\left(y_{k+1}\right)}, & \mathrm{if}\;p_{k}\notin \partial E^{\varepsilon } \end{array}\right.$$

where $r_{k}=min_{p\in \partial E}||p-p_{k}\left|\right|$ is the closest distance from point $p_{k}$ to explored region boundary ∂E, $p_{k+1}\in \partial B\left(p_{k},r_{k}\right)$ and $y_{k+1}\in B\left(p_{k},r_{k}\right)$ are sampled from uniform probabilities $p^{\partial B(p_{k},r_{k})}=U\left(\partial B\left(p_{k},r_{k}\right)\right)$ and $p^{B(p_{k},r_{k})}=U\left(B\left(p_{k},r_{k}\right)\right)$, respectively. Because we deal with the problem in the 2D world, then the above estimator becomes:

(6) $$\hat{\varphi }\left(p_{k}\right):=\left\{\begin{array}{cc} g(\bar{p_{k}}), & \mathrm{if}\;p_{k}\in \partial E^{\varepsilon }\\ \hat{\varphi }\left(p_{k+1}\right)+\pi r_{k}^{2}f\left(y_{k+1}\right)G^{B(p_{k},r_{k})}, & \mathrm{if}\;p_{k}\notin \partial E^{\varepsilon } \end{array}\right.$$

Essentially, as we also see in Figure 1b, the algorithm determines a random walk starting at point $p_{0}$, finds the largest ball $B(p_{0},r_{0})$ contained in E, picks a point $p_{1}\in \partial B\left(p_{0},r_{0}\right)$ and another one $y_{1}\in B\left(p_{0},r_{0}\right)$, and continues the same process with starting point $p_{1}$. The algorithm terminates when $p_{k}$ is in the region $\partial E^{\varepsilon }$, e.g., $r_{k}<\varepsilon$, and then we get the Dirchlet value $g(\bar{p_{k}})$. For N>0 random walks, the solution of the Poisson equation is:

(7) $$\varphi \left(p_{0}\right)=\frac{1}{N}\sum_{i=1}^{N}\hat{\varphi }\left(p_{0}\right)$$

Algorithm 1 involves the process that we discussed above. It should be noted that in line 7, the function *closestPointOfBoundary* returns the closest point $\bar{p_{i}^{\prime }}\in \partial E$ from a point $p_{i}^{\prime }\in E$ and the distance $r_{i}$ of these points. Also, in lines 11 and 13, we call the sampling functions over a ball. The function *samplePointInBall* samples a point $y_{i}∼U\left(B\left(p_{i}^{\prime },r_{i}\right)\right)$, and *samplePointOnBallBoundary* samples a new point $p_{i}^{\prime }∼U\left(\partial B\left(p_{i}^{\prime },r_{i}\right)\right)$.
**Algorithm 1** WalkingOnSpherePoissonEquation$\left(p_{0},E,N,\varepsilon \right)$
   **Input:** Starting point $p_{0}$, explored region E, *N* number of walks, ε termination parameter.   **Output:** The solution of the Poisson Equation $\varphi (p_{0})$                            ▹ Initialize the Poisson’s solution1:$\varphi (p_{0})\leftarrow 0$                                  ▹ Start the process2:i←13:**while** i≤N **do**4:    $p_{i}^{\prime }\leftarrow p_{0}$5:    $\varphi (p_{i}^{\prime })\leftarrow 0$                                 ▹ Start the *i*-th walk6:    **while** true **do**                       ▹ Find the closest point from point $p_{i}^{\prime }$ to ∂E7:        $\bar{p_{i}^{\prime }},r_{i}\leftarrow$ closestPointOfBoundary$\left(p_{i}^{\prime },E\right)$                         ▹ Check if $p_{i}^{\prime }$ is closed enough to the ∂E8:        **if** $r_{i}<\varepsilon$ **then**9:           **break**10:        **end if**                         ▹ Sample point in the ball the $B(p_{i}^{\prime },r_{i})$11:        $y_{i}\leftarrow$ samplePointInBall$\left(p_{i}^{\prime },r_{i}\right)$                               ▹ Add the source value12:        $\varphi \left(p_{i}^{\prime }\right)\leftarrow \varphi \left(p_{i}^{\prime }\right)+\pi r_{i}^{2}f\left(y_{i}\right)G^{B(p_{i}^{\prime },r_{i})}\left(p_{i}^{\prime },r_{i}\right)$                     ▹ Sample new point on the boundary $\partial B(p_{i}^{\prime },r_{i})$13:        $p_{i}^{\prime }\leftarrow$ samplePointOnBallBoundary$\left(p_{i}^{\prime },r_{i}\right)$14:        i←i+115:    **end while**                              ▹ Add the Dirichlet Value16:    $\varphi \left(p_{0}\right)\leftarrow \varphi \left(p_{0}\right)+\varphi \left(p_{i}^{\prime }\right)+g\left(\bar{p_{i}^{\prime }}\right)$17:**end while**                             ▹ Caclulate the Mean Value18:$\varphi \left(p_{0}\right)\leftarrow \frac{1}{N}\varphi \left(p_{0}\right)$19:**return:** $\varphi (p_{0})$

**Figure 2:**
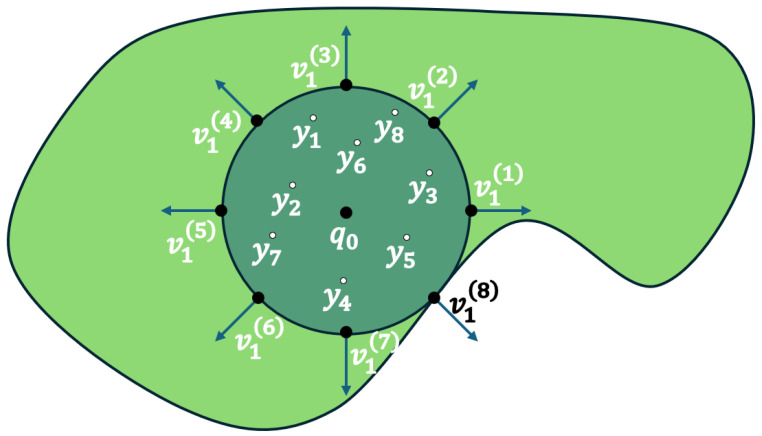
The order of outward unit vectors $\nu_{1}$ on the $\partial B(p_{0},r_{0})$.

Since we analyzed how to find the solution of the Poisson equation, now we will present how to calculate its gradient $\nabla \varphi (p_{0})$. According to [26], a basic WoS estimator for calculating the gradient of the solution $\varphi (p_{0})$ for the ball $B(p_{0},r_{0})$ is shown below:

(8) $$\nabla \varphi \left(p_{0}\right)=\frac{1}{N_{G}}\left(\sum_{i=1}^{N_{G}}\nabla \hat{\varphi }\left(p_{0}\right)+\pi r_{0}^{2}f\left(y_{i}\right)\nabla G^{B(p_{0},r_{0})}\left(p_{0},y_{i}\right)\right),$$

where $\nabla \hat{\varphi }\left(p_{0}\right)=\frac{2}{r_{0}}\varphi \left(p_{1}\right)\nu \left(p_{1}\right)$. Essentially, in (8), we sample $N_{G}$ uniform points on the boundary of the largest sphere in E and calculate the solution $\varphi (p_{1})$ for each point $p_{1}$ with Algorithm 1. Notice that instead of sampling randomly in the $\partial B(p_{0},r_{0})$, we rather use a deterministic method so that each point maintains an approximately equal geodesic distance from its neighbors, as we present in Figure 2. In this manner, we do not violate the uniform distribution properties. Furthermore, we uniformly sample in the ball $B(p_{0},r_{0})$$N_{G}$ points $y_{1}$ to calculate the Green term of our estimator. In Algorithm 2, we demonstrate the calculation process of the gradient. The extra function that is added to the algorithm is the *outwardVectorPoints* that returns an array $p_{i}$ of dimension $R^{N_{G}\times 2}$ with the radial directions of points sampled as we said before.
**Algorithm 2** WalkingOnSphereGradientSolution$\left(p_{0},E,N_{G},N,\varepsilon \right)$
   **Input:** Starting point $p_{0}$, explored region E, $N_{G}$ number of samples for calculating the gradient of boundary term, *N* number of walks, ε termination parameter.  **Output:** The gradient of the solution of the Poisson Equation $\nabla \varphi (p_{0})$                    ▹ Initialize the gradient Poisson’s solution1:$\nabla \varphi \left(p_{0}\right)\leftarrow {\left[0,0\right]}^{T}$                ▹ Initialize the outward vector points $p_{1}\in R^{N_{G}\times 2}$2:$\bar{p_{0}},r_{0}\leftarrow$ closestPointOfBoundary$\left(p_{0},E\right)$3:$p_{1}\leftarrow$ outwardVectorPoints$\left(p_{0},r_{0},N_{G}\right)$                         ▹ Calculate Gradient Solution4:i←15:**while** $i\leq N_{G}$**do**6:    $p_{i}\leftarrow p_{1}\left[i\right]$7:    $\varphi (p_{i})=$ WalkingOnSpherePoissonEquation$\left(p_{i},E,N,\varepsilon \right)$                     ▹ Sample point in the ball the $B(p_{0},r_{0})$8:    $y_{i}\leftarrow$ samplePointInBall$\left(p_{0},r_{0}\right)$                   ▹ Add the *i*-th gradient of boundary term9:    $\nabla \varphi \left(p_{0}\right)\leftarrow \nabla \varphi \left(p_{0}\right)+\frac{2}{r_{0}}\varphi \left(p_{i}\right)\nu \left(p_{i}\right)+\pi r_{0}^{2}f\left(y_{i}\right)\nabla G^{B(p_{0},r_{0})}\left(p_{0},y_{i}\right))$10:**end while**                          ▹ Caclulate the gradient11:$\nabla \varphi \left(p_{0}\right)\leftarrow \frac{1}{N_{G}}\nabla \varphi \left(p_{0}\right)$12:**return:** $\nabla \varphi (p_{0})$

**Remark 2.**  *The complexity of the WoS algorithm depends on the number of sampled points as well as on the termination factor ε that effects the number of steps needed to reach the $\partial E^{\varepsilon }$. According to [35], the typical number of steps for a single walk is O(ln(1/ε)). Consequently, the time complexity of Algorithm 2 is $O(N_{G}\cdot N\cdot \ln\left(1/\varepsilon \right))$, which can be computationally expensive. In order to resolve this, we modify the algorithm by introducing a dynamic, vectorized implementation. This approach allows the execution of multiple parallel walks, approaching O(ln(1/ε)) step complexity, and improves the overall computational efficiency of the WoS algorithm. The dynamic modification of the algorithms is not complicated.
*

## 4. Implementation of the Exploration Process

In this section, we discuss the process followed to address the exploration problem. More specifically, we present the controller architecture and we provide the safety and completeness guarantees of the proposed control law.

### 4.1. Control Architecture

**Figure 3:**
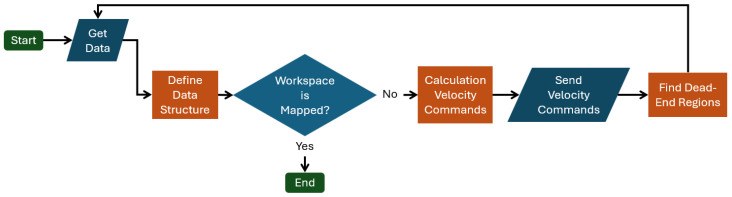
Flowchart of the exploration method.

In Figure 3, we present the main steps of the proposed method. First, we gather data from the range sensor—set $S(p;R_{max})$)—with the map of the workspace and the position of the agent. The agent updates this data as it moves in the workspace. Afterwards, in the block *Define Data Structure*, we construct the explored space and the *Hybrid Visibility Graph*, and we determine the boundary value conditions and source function. Then, we check if the map is complete. If it is not, we calculate in the block *Calculation Velocity Commands* the velocity commands of the agent through the WoS algorithm and execute it. As the agent moves, in the block *Find Dead-End Regions*, we inspect if some region is Dead-End, e.g., a region that the agent has explored and does not need to traverse it again. If the dead-end algorithm finds a region in block *Define Data Structure*, it removes it from the explored set E. So, the above exploration process is recursive and stops when the map is fully explored.

### 4.2. Velocity Control Law

The velocity control law is given by:

(9) $$u=-\frac{\nabla \varphi (p)}{||\nabla \varphi (p)||+\eta }$$

where η>0 is a small positive constant so as to prevent the discontinuity when ∇φ→0. The control law is a normalized vector w.r.t. the x-coordinate and y-coordinate, i.e., $u={\left[\begin{array}{cc} u_{x}, & u_{y} \end{array}\right]}^{T}$.

As we mentioned previously, the agent moves in two ways: it rotates to reduce the orientation error $e_{\theta }=\theta_{d}-\theta$, where $\theta_{d}=atan2\left(\frac{u_{y}}{u_{x}}\right)$, and as $e_{\theta }\to 0$, the agent moves forward, following the linear and angular velocity commands defined as $v_{l}=K_{l}\cdot s$ and $v_{a}=K_{a}\cdot e_{\theta }$, where $K_{l},K_{a}>0$ are the gains of linear and angular velocity and $s=S_{a}\left(d\left(p,\partial E\right)\right)$, with α>0, where $S_{\alpha }\left(.\right)$ is a bump function that asymptotically approaches zero as the agent approaches the boundary. A candidate function is [22]:

(10) $$S_{\alpha }=\left\{\begin{array}{cc} 1, & x>\alpha \\ 3{\left(\frac{x}{a}\right)}^{2}-2{\left(\frac{x}{a}\right)}^{3}, & 0\leq x\leq a\\ 0, & x<0 \end{array}\right.$$

Notice that whenever the minimum Euclidean distance d(p,∂E)<α, the agent slows down.

### 4.3. Structure of Explored Region and Definition of Hybrid Visibility Graph

The boundary of the explored space ∂E involves disjoint continuous curves of free and occupied cells, $\partial E_{f}$ and $\partial E_{o}$, respectively, which form a polygon in 2D space, defined by a connected undirected graph $G_{E}=\left(V_{E},E_{E},w_{E}\right)$, with $V_{E}=\left\{v_{01},v_{02},\ldots ,v_{0n_{0}},v_{11},\ldots ,v_{N_{h}n_{h}}\right\}$ denoting the vertices of the polygon, where $n_{0}$ is the number of vertices of the exterior boundary of the polygon, $n_{i},i=1,\ldots ,n_{h}$ is the number of vertices in the interior, $E_{E}=\left\{\left(v_{01},v_{02}\right),\left(v_{02},v_{03}\right),\ldots ,\left(v_{0n_{0}},v_{01}\right),\left(v_{11},v_{12}\right),\ldots ,\left(v_{N_{h}n_{h}},v_{N_{h}1}\right)\right\}$ is the edge list of the graph, and $w_{E}=\left\{w_{01},w_{02},\ldots ,w_{0n_{0}},w_{11},\ldots ,w_{N_{h}n_{h}}\right\}$ denotes the weight’s set of the graph’s edges. Notice that the distances of the edges are the same and equal to the resolution of the map $m_{r}$. Beyond the aforementioned graph, we define a set of points $Q_{m}=\left\{p_{01},p_{02},\ldots ,p_{0n_{0}},p_{11},\ldots ,p_{N_{h}n_{h}}\right\}$ that belong to the middle of the edges, with the total number of them to be $N_{V_{E}}$. Some of them are on the free boundary $\partial E_{f}$ and others on the occupied $\partial E_{o}$, denoted as $Q_{mf},Q_{mo}\subset Q_{m}$, respectively. Also, the sets $Q_{mf}={⋃}_{i\in I_{f}}Q_{mf_{i}}$ and $Q_{mo}={⋃}_{i\in I_{o}}Q_{mo_{i}}$ involve the subsets of the free and occupied middle points of the discretized curves.

**Figure 4:**
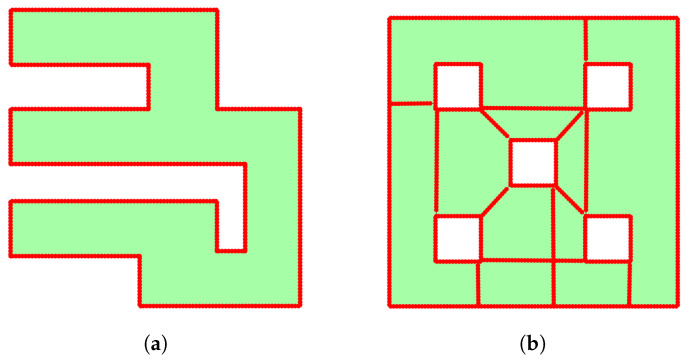
Examples of HVG form. (**a**) Workspace without interior obstacles and (**b**) workspace with interior obstacles. The red points are the vertices of HVG.

Now, in order to solve the problem of the flat gradient, we first have to define the HVG. The HVG $G_{H}=\left(V_{H},E_{H},w_{H}\right)$ is a connected undirected graph. Its vertices $V_{H}$ involve the set of middle points $Q_{m}$ and the set of points forming minimum-length edges between exterior and interior obstacles, denoted by $Q_{m}^{\prime }$. In Algorithm 3, we introduce the steps for constructing this graph. We initialize the $G_{H}$ by adding the middle points $Q_{m}$, edge list of this set, and the edge’s weight such as in the $G_{E}$ (lines 1–3). We use the boolean function *isInvolveObstacles* to check if the $G_{E}$ involves inner obstacles or holes (lines 4–6). If the explored space has no holes, the algorithm returns the pre-initialized graph. However, if holes are present, we begin the process of finding the minimum-length edges, connecting each exterior and interior obstacle. First of all, we separate the holes from the explored region and store them in a set $H=\{H_{0},\ldots ,H_{M_{h}}\}$ where $H_{0}$ represents the exterior boundary of the explored space and $H_{i},i=1,\ldots ,H_{M_{h}}$ the boundary of interior obstacles. This separation is handled by the function *getObstaclesSet*, a property of structure $G_{E}$ (line 7). After separating the obstacles, we proceed with the connection process as detailed in the lines 8–21. This involves taking two obstacles and using the function *findMinimumVisibleEdge* to locate points that make the minimum-length edge and not cross the occupied cells. If the above properties are true, the boolean variable *isVisible* will be equal to the true value (line 12). If *isVisible* is false, we continue the process; otherwise, we start the process by adding this edge to the $G_{H}$ (lines 13–15). Before adding the edge to the graph, the function *discretizeEdge* discretizes it into smaller segments, each with a distance of $m_{r}$ (line 16). Finally, we add the newly generated vertices $q_{m}^{\prime }\subset Q_{m}^{\prime }$ and edges to $G_{H}$ (lines 17–19). At the end of the process, the algorithm returns the final HVG (line 22). In Figure 4, we present some results of this algorithm, one workspace without (Figure 4a) and one with (Figure 4b) obstacles.

**Remark 3.**  *The time and memory complexity of the aforementioned process depend on the number of explored interior obstacles. As the number of holes increases in the explored region, our approach slows down when extracting the new state of the agent. To address this, we apply techniques like the Dead-End algorithm (Section 4.5), which restricts the number of obstacles within the exploration space.
*

The extraction of this graph is helpful because we have to make an approximation of the geodesic distance from any point in E to free continuous curves. In that way, we can determine the source function that attracts the agent from the dead lock regions to the free space.
**Algorithm 3** DefineHybricVisibilityGraph$\left(G_{E},Q_{m},N_{V_{E}}\right)$
   **Input:** The explored region graph $G_{E}$, the middle point $Q_{m}$ of the edges in the set $E_{E}$, $N_{V_{E}}$ the number of middle points.   **Output:** The HVG $G_{H}$                                  ▹Initialize the $G_{H}$1:$G_{H}.V_{H}\leftarrow$ addnodes$\left(Q_{m}\right)$2:$G_{H}.E_{H}\leftarrow$ addedges$\left(Q_{m},{\left[Q_{m}{\left(2:end,:\right)}^{T},Q_{m}{\left(1,:\right)}^{T}\right]}^{T}\right)$3:$G_{H}.w_{H}\leftarrow$ addweight$(m_{r}\cdot$ ones$\left(N_{V_{E}},1\right)$                  ▹Check if explored region doesn’t involve obstacles4:**if not**(isInvolveObstacles$\left(G_{E}\right)$) **then**5:    **return:** $G_{H}$6:**end if**            ▹Get the obstacles set $H=\{H_{0},\ldots ,H_{M_{h}}\}$ from the explored region7:$H,M_{h}\leftarrow G_{E}.$getObstaclesSet()             ▹Connect each obstacle with the minimum-length visible edge8:**for** *i* in **range**$\left(1,M_{h}\right)$ **do**                             ▹Get the *i*-th element of H9:    $h_{i}\leftarrow H\left\{i\right\}$10:    **for** *j* in **range**$\left(2,M_{h}+1\right)$ **do**                            ▹Get the *j*-th element of H11:        $h_{j}\leftarrow H\left\{j\right\}$       ▹Find the points of minimum-length visible edge of *i*-th and *j*-th obstacle12:        $v_{i},v_{j},isVisible\leftarrow$ findMinimumVisibleEdge$\left(h_{i},h_{j}\right)$                ▹Check if the point $v_{i}$ is in the field of view of point $v_{j}$13:        **if not**(isVisible) **then**14:           **continue**15:        **end if**       ▹Discretize the resulting edge into points set $q_{m}^{\prime }$ with distance $m_{r}$ and total number $N_{q_{m}^{\prime }}$16:        $q_{m}^{\prime },N_{q_{m}^{\prime }}\leftarrow$ discretizeEdge$\left(v_{i},v_{j},m_{r}\right)$                        ▹Add the new elements into the $G_{H}$17:        $G_{H}.V_{H}\leftarrow$ addnodes$\left(q_{m}^{\prime }\right)$18:        $G_{H}.E_{H}\leftarrow$ addedges$\left(q_{m}^{\prime },{\left[q_{m}^{\prime }{\left(2:end,:\right)}^{T},q_{m}^{\prime }{\left(1,:\right)}^{T}\right]}^{T}\right)$19:        $G_{H}.w_{H}\leftarrow$ addweight$\left(m_{r}\cdot J_{N_{q_{m}^{\prime }}}\right)$20:    **end for**21:**end for**                           ▹Finally, return the HVG $G_{H}$22:**return:** $G_{H}$

### 4.4. Dirichlet Boundary Value and Source Function

Having defined the explored polygon $G_{E}$ and the sets of middle points, let us define the boundary values (2). We determine the weight of each edge of the graph $G_{E}$ equal to a Dirichlet boundary value $g(p_{m})$, where $p_{m}\in Q_{m}$.

**Figure 5:**
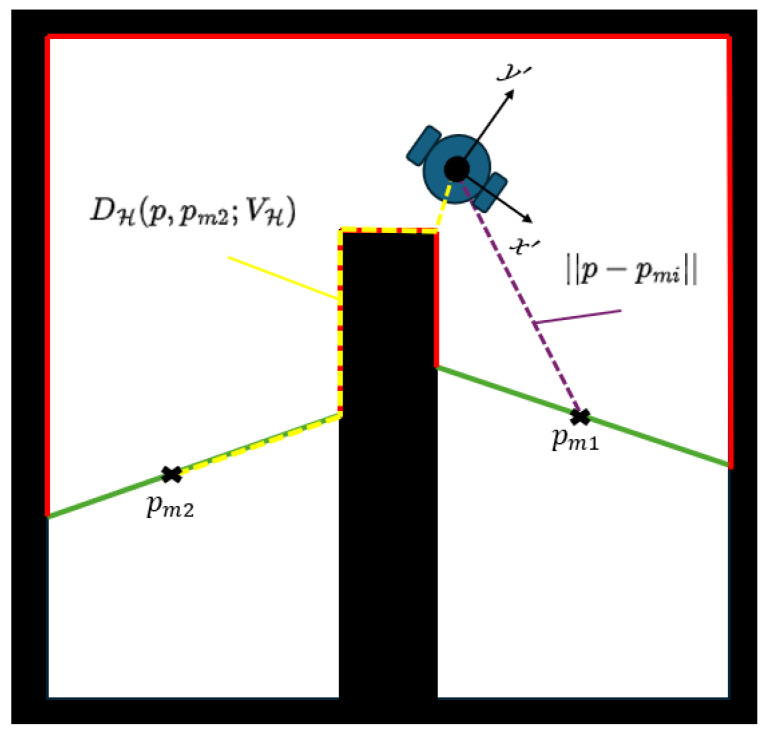
An example where we see how the agent calculates the distance to the free frontiers (green lines). In the first frontier with middle point $p_{m1}$, we calculate the Euclidean distance (purple dashed line) and the other $p_{m2}$ with $D_{H}\left(p,p_{m2};V_{H}\right)$ (yellow dashed line).

To determine the closest free curve $\partial E_{fi}$ from the current agent’s position *p*, which is assigned attractive, let $p_{mi}\in Q_{mf_{i}}$ be a point that belongs to the continuous free curve $\partial E_{fi}$ and is located in the middle of this curve. The distance is calculated by:

(11) $$d_{g}\left(p,p_{mi}\right)=\left\{\begin{array}{cc} ||p-p_{mi}\left|\right|, & p_{mi}\in V\left(p;E\right)\\ D_{H}\left(p,p_{mi};V_{H}\right) & p_{mi}\notin V\left(p;E\right) \end{array}\right.$$

where V(p;E)={z∈E|(1−t)z+tz∈E,∀t∈[0,1]} is the visibility region of the agent in the explored space E and $D_{H}\left(p,p_{mi};V_{H}\right)=d\left(p,V_{H}\right)+d_{G_{H}}\left(p_{m}^{\prime },p_{mi}\right)$ is the sum of the minimum distance $d(p,V_{H})$ from the agent’s position to the set $V_{H}$ and the distance $d_{G_{H}}\left(p_{m}^{\prime },p_{mi}\right)$, with $p_{m}^{\prime }=argmin_{p_{m}^{\prime }\in V_{H}}||p-p_{m}^{\prime }\left|\right|$, which is the shortest path from $p_{m}^{\prime }$ to $p_{mi}$ based on the HVG. An example of this metric is shown in Figure 5. The aforementioned distance indicates that if the free curve $\partial E_{fi}$ lies in the field of view, we calculate the Euclidean distance. If it is not, then we take the distance from the HVG. We follow this policy for the shortest point, because the Euclidean distance is not a proper choice to estimate the distance from one point to another in non-convex regions [36].

**Remark 4.**  *
The selection of agent’s free frontier is not governed by a utility function. Thus, optimal path determination is not guaranteed. Consequently, if the agent proceeds in a sub-optimal direction, it will explore and will approach the next closest free frontier.
*

So, the attractive curve is calculated by $\partial E_{f}^{a}=argmin_{p_{mi}\in \partial E_{fi}}\left(d_{g}\left(p,p_{mi}\right)\right)$, and the rest of the free curves, which are set as repulsive, are denoted as $\partial E_{f}^{o}$. Since we have defined the attractive and occupied regions, we assign the boundary value function as follows:

(12) $$g\left(p\right)=\left\{\begin{array}{cc} -k_{f}, & p\in \partial E_{f}^{a}\\ k_{o}, & p\in \partial E_{f}^{o}\cup \partial E_{o} \end{array}\right.$$

where $k_{f},k_{o}>0$ are constant variables.

**Remark 5.**  *
The policy selection of a single free frontier as attractive serves to create a potential field, leading agents from anywhere in the explored region towards it effectively.
*

**Figure 6:**
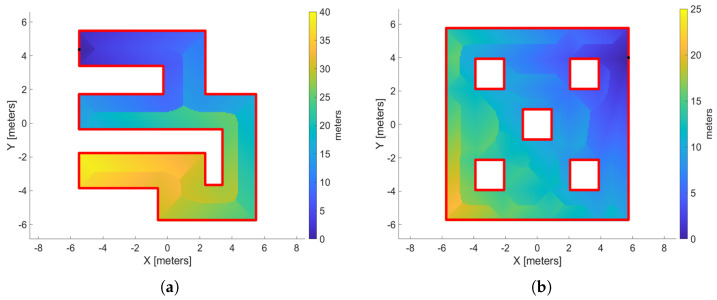
Two workspace examples that exhibit how the distance metric to the source point is distributed in those regions. (**a**) Workspaces without interior obstacles and (**b**) workspace with interior obstacles. The black dot is the source point.

As we chose the attractive free curve, the middle point $p_{mi}\in \partial E_{f}^{a}$ is defined as the source point denoted by $p_{s}$. In a workspace with obstacles, we connect the point $p_{s}$ to any obstacles within its field of view, adding these new edges to $G_{H}$. In this manner, we improve the estimation of geodesic distances to $p_{s}$, i.e., the function $D_{H}\left(y,p_{s};V_{H}\right)$. The choice of the source function of point $p_{s}$ will be based on the attributes of the above distance metric. In Figure 6, we have two workspaces—without (Figure 6a) and with (Figure 6b) obstacles—and see the distribution of the distance metric in these platforms with respect to the source point $p_{s}$, denoted by the black dot.

As we expected, boundaries are created in the explored space (like the Voronoi Diagram), which separate the regions close to the source point and those far from it. Thus, we have to enhance the regions which are close to the source point and reduce the influence of the distant regions so that it promotes the sampling of outward vectors ν of ∇φ to lead the agent toward the attractive region $\partial E_{f}^{a}$. To achieve, this we proposed the following exponential source function:

(13) $$f\left(p;p_{s}\right)=-{\left(1-\frac{D_{H}\left(p,p_{s};V_{H}\right)}{\Pi (\partial E)}\right)}^{k_{s}}$$

where Π(∂E) is the perimeter of the explored space boundary and $k_{s}$ is a fixed positive gain. Hence, the function *f* attains values in [−1,0], which means that if the agent is close enough to the source point, then f(.)→−1, and when it is far away, then f(.)→0. So, the value of $f(y_{i})$ with $y_{i}$ denoting the sampling point we get in the process of the WoS algorithm approximates value −1 when $D_{H}\left(y_{i},p_{s};V_{H}\right)\to 0$. As a result, the solution (8) amplifies the vectors $\nu (p_{i})$, thus leading the agent to the free frontier.

**Figure 7:**
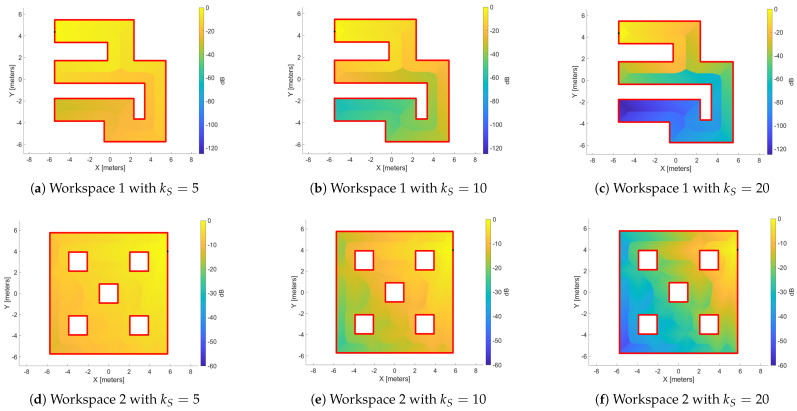
The distribution of the source value with different values of variable $k_{S}=5,10,20$. (**a**–**c**) present a workspace without obstacles and (**d**–**f**) a workspace with interior obstacles. The black dot denotes the source point $p_{s}$, and the source values is in dB.

**Remark** **6.** *
The source function is derived heuristically, based on the distribution of the distance function $D_{H}$ (Figure 6) with respect to the source point $p_{s}$. The primary goal of this design is to enable the agent, when situated in a region distant from point $p_{s}$, to escape that area and be led toward the free frontier. Figure 7 illustrates the distribution of the source values in decibel ($20log(|f\left(p;p_{s}\right)|)$) to the the workspaces of Figure 6 for different values of variable $k_{s}\left(=5,10,20\right)$. As observed in the heat maps, increasing the variable $k_{s}$ reduces the influence of distant regions, thereby achieving our stated objective.
*

### 4.5. Dead-End Region Algorithm

During the WoS algorithm, walkers may enter regions containing only occupied cells with a single escape path. This leads to increased computational and memory complexity, as walkers must traverse substantial distances to reach the explored region’s boundary. As a result, this does not offer further exploratory value. To eliminate this, our exploration process introduces an algorithm, based on [24], that removes these explored, dead-end regions, preventing the agent from re-traversing them.

The identification method of dead-end regions is handled within the *Find Dead-End Regions* block. The process is as follows: Let $t_{I_{c}}$ be the current exploration time step at position $p_{c}$, where $I_{c}$ presents the number of current iteration number of exploration process. Up to this point, the agent has traversed a path $P(t_{c})$ and stored all scanned regions at each time step in a set denoted as $S_{T}=\left\{S_{1},S_{2},\ldots ,S_{I_{c}}\right\}$, where $S_{i}=S\left(p\left(t_{i}\right);R_{max}\right)$ for $i=1,\ldots ,I_{c}$ with $t_{1}$ being the initial exploration time step. At each exploration time step, the dead-end method checks all regions within $S_{T}$ to determine if they are dead-end. If the method identifies regions as dead-end and the agent is not currently within them, these regions are removed from $S_{T}$ and stored in a separate set, $S_{D}$. So, in the *Define Data Structure* block, it subtracts these regions from the overall exploration space. To optimize the algorithm’s time performance, the removal of dead-end regions is not executed at every exploration step. Instead, we perform this subtraction every $N_{D}>0$ iterations, as the process can become computationally slow if executed too frequently or if the set $S_{D}$ contains a large number of regions.

**Remark 7.**  *The aforementioned process can become computationally slow if executed too frequently or if the set $S_{D}$ contains a large number of regions. As a result, the agent can be stopped in order to calculate the new exploration region or be trapped if the cutting is abrupt. To optimize our algorithm’s time performance and ensure the agent to be distant from the reducing region, the subtraction of dead-end regions is not executed always at every exploration step, but it performs every $N_{D}>0$ iterations.
*

**Figure 8:**
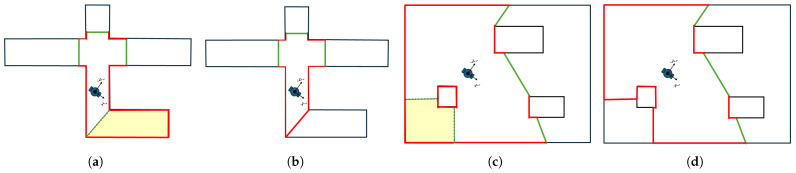
Two examples of dead-end exploration region removing, presented with yellow area. In (**a**,**b**) the removed region doesn’t involve occupied boundaries of interior obstacles, whereas (**c**,**d**) involve them. Also, (**a**,**c**) are in the time step before removing and (**b**,**d**) after. The red lines denote the occupied boundaries of exploration space and green lines the free one.

For a region $S_{i}\in S_{T}$ to be labeled as dead-end and then removed from exploration space, its boundaries must contain a specific number of continuous free boundary curves. This number is related to the number of occupied boundaries from known interior obstacles within region $S_{i}$. Specifically, a region is considered as dead-end if it possesses one plus the number of occupied boundary obstacles as continuous free boundaries. In Figure 8, we introduce this concept with two examples of removed regions (yellow area). In the first example (Figure 8a,b), the removed region contains no occupied boundaries from any obstacle. So, it is labeled as dead-end, since it includes only one continuous free boundary curve. Similarly, in the second example (Figure 8c,d), the region contains a continuous occupied boundary curve from an obstacle. Therefore, it requires two continued free boundary curves to be identified as dead-end.

### 4.6. Safety and Completeness of Exploration

**Figure 9:**
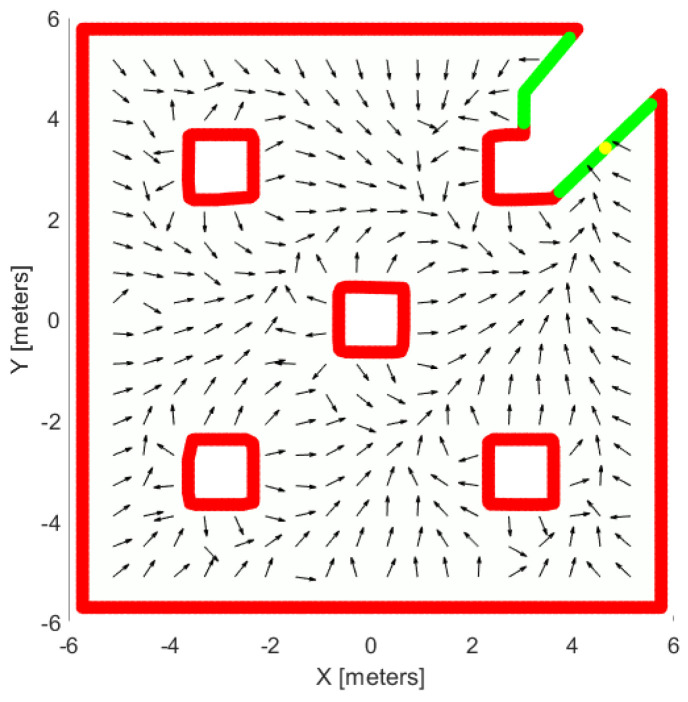
An example of a vector field made from our exploration controller. The red lines are the occupied cells, the green lines are the free frontiers, the yellow point is the source point and black vectors show the orientation of the field.

The proposed exploration process is safe and complete. Based on the proposed controller u(p), and the definition of the Dirichlet boundary conditions as well as of the source function, the agent always is repulsed by the occupied boundary of the explored region. Moreover, owning to the term *s* in the linear velocity command, the agent never collides with the boundary, since d(p,E)→0 leads to $v_{l}\to 0$. Regarding the completeness, if we tune properly the exploration parameter for boundary conditions and source function values, the agent is led to the pre-defined free regions and there is no deadlock situation in the flat areas. In Figure 9, we illustrate a workspace with obstacles and the potential field created by the proposed controller, which leads the agent towards the free frontier without colliding with the obstacles from anywhere in the workspace.

The aforementioned solution is heuristic, as the agent occasionally demonstrates regressions in its exploration process. However, by tuning the exploration parameters and incorporating a lot of walkers within the Walk on Spheres (WoS) method, the agent is able to move away from regions with flat gradients and expand the explored domain.

## 5. Tools for Real Experiment

**Figure 10:**
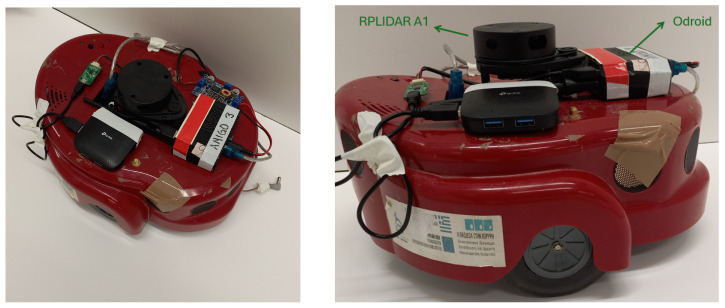
The AmigoBot unit (**Left**) and its equipment (**Right**).

In this project, we test our exploration process with a real experiment, utilizing a differential drive AmigoBot [37] (see Figure 10). The AmigoBot is a mobile robot appropriate for motion planning applications, which can be monitored using the ROS framework and Matlab. For our experiment, the AmigoBot is integrated with an RPLIDAR A1 laser scanner, developed by SLAMTEC (Shanghai, China), and an odroid processing unit embedded with Ubuntu 20.04.6 LTS (Focal Fossa), 2GB RAM, and ARMv7 processor. Also, the MATLAB environment version 2023b is installed in our laptop with the operating system Ubuntu 20.04, 8GB RAM, and Intel(R) Core(TM) i7-7500U processor. The ROS Noetic and ARIA library for gathering sensor data and monitoring the AmigoBot are installed in both systems.

The robot’s position is obtained from the gmapping package version 1.4.2, which is a library of the ROS Noetic framework that provides laser-based SLAM. The AmigoBot is assumed to be a disk-shaped robot with radius 0.3 m with the LiDAR sensor mounted at its center with maximum range 3 m.

**Figure 11:**
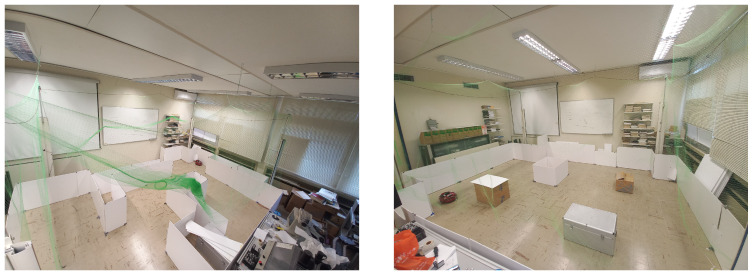
Real-world maps: (**Left**) Labyrinth and (**Right**) workspace with obstacles.

The workspace of our experiment was in the Autonomous Robotics Lab of the Department of Electrical & Computer Engineering at the University of Patras. We built within a space two different maps to test our method, one labyrinth and another that involves only obstacles as we see in Figure 11.

## 6. Results

In this section, we present the results of the proposed method in three studies. First, we present a simulation in Matlab, where we demonstrate the safety and completeness of our algorithm. In the second part, we present a real-world experiment with the AmigoBot unit. Finally, in the third part, we introduce a comparison study of our method’s results with other exploration methods. In Table 1, we define the parameter values of the exploration process for the three parts.

Furthermore, to evaluate our algorithm’s efficiency, we use three metrics: (1) the number of exploration steps, (2) the total path length, and (3) the overall exploration time. Moreover, for each iteration, we also report the average time required to (i) collect and process data, (ii) calculate the velocity command with the WoS algorithm, (iii) move the agent to the new state, and (iv) discover the dead-end regions. Finally, we provide the average iteration time both with and without the robot’s movement.

**Figure 12:**
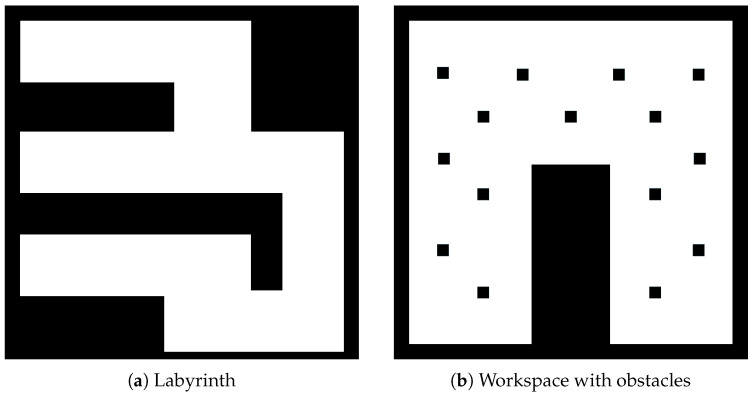
Exploration maps for part A with Matlab.

**Figure 13:**
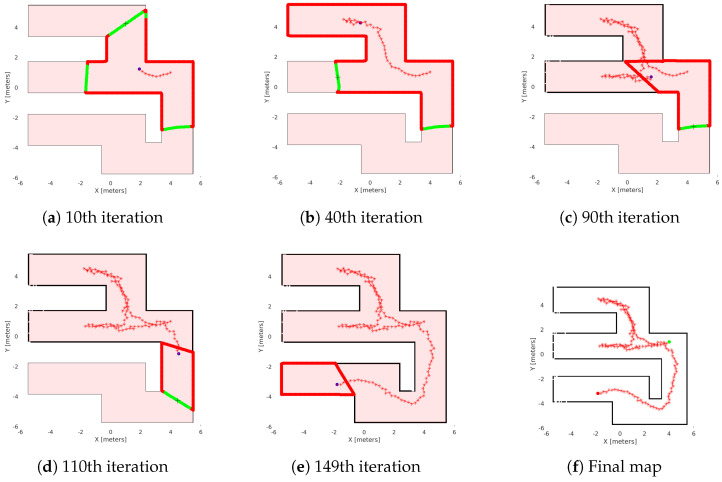
Exploration process of the Matlab simulation in the labyrinth. (**a**–**e**) depict the exploration process at various stages of the method’s execution, with (**f**) representing the agent’s complete explored map. The green lines are the free frontiers, red lines are the occupied frontiers, the black cross point is the source point, and the starred red points denote the exploration path of the agent denoted with blue dot.

**Figure 14:**
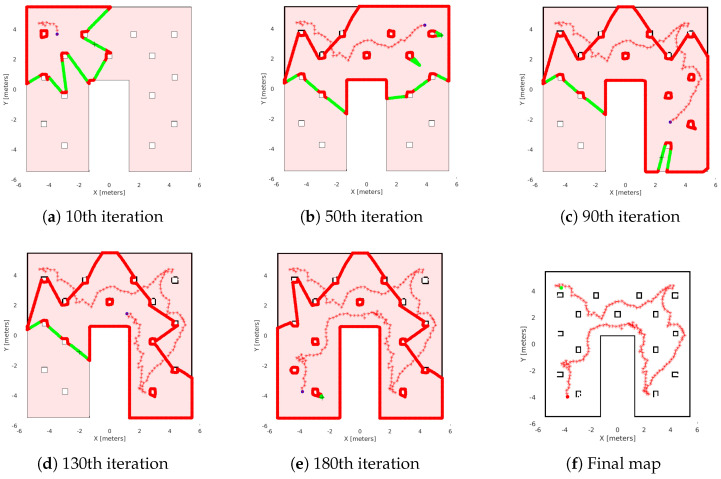
Exploration process of the Matlab simulation in the workspace with obstacles. (**a**–**e**) depict the exploration process at various stages of the method’s execution, with (**f**) representing the agent’s complete explored map. The green lines are the free frontiers, red lines are the occupied frontiers, the black cross point is the source point, and the starred red points denote the exploration path of the agent denoted with blue dot.

In the first study, we present two simulations. We have two 11 m × 11 m maps to explore, as we see in Figure 12. The first map (see Figure 12a) is a labyrinth and the second one is a workspace with obstacles (see Figure 12b). In the results, we omit the time of the agent’s movement to examine only the average time of exploration algorithms. In Figure 13, we see the exploration process of the mobile agent into the labyrinth and in Figure 14 the workspace with obstacles. In Table 2, we introduce the results of the first part. For these simulations, we use the same hardware as in the real experiment.

**Figure 15:**
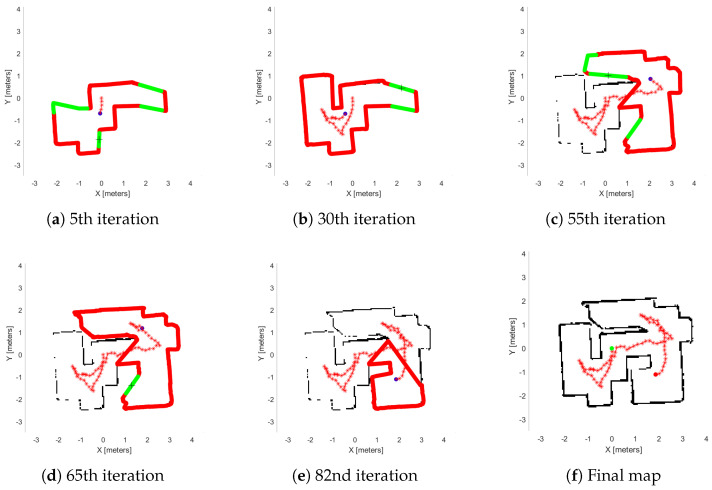
Real-world exploration process in the labyrinth. (**a**–**e**) depict the exploration process at various stages of the method’s execution, with (**f**) representing the agent’s complete explored map. The green lines are the free frontiers, red lines are the occupied frontiers, the black cross point is the source point, and red star points denote the exploration path of the agent denoted with blue dot.

**Figure 16:**
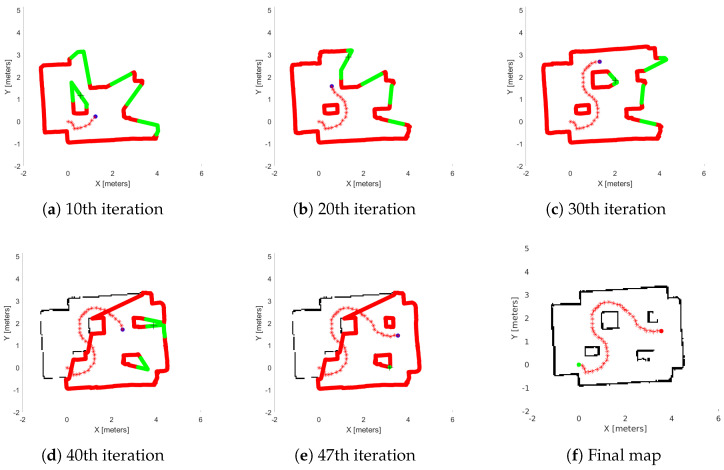
Real-world exploration process in the Workspace with obstacles. (**a**–**e**) depict the exploration process at various stages of the method’s execution, with (**f**) representing the agent’s complete explored map. The green lines are the free frontiers, red lines are the occupied frontiers, the black cross point is the source point, and the starred red points denote the exploration path of the agent denoted with blue dot.

Next, we demonstrate two real-world experiments. As previously mentioned, we use an AmigoBot unit for our experiment and as a communication tool, the ROS framework. We constructed two maps similar to the first study, as we can see in Figure 11. In Figure 15, we see the exploration process of the AmigoBot unit into the labyrinth and in Figure 16 in the workspace with obstacles. The corresponding experimental results are summarized in Table 3. In this video https://youtu.be/EmfcYx3QZMo accessed on 12 June 2025, we present the simulation of the labyrinth of the first part and the workspace with obstacles of the second part.

**Figure 17:**
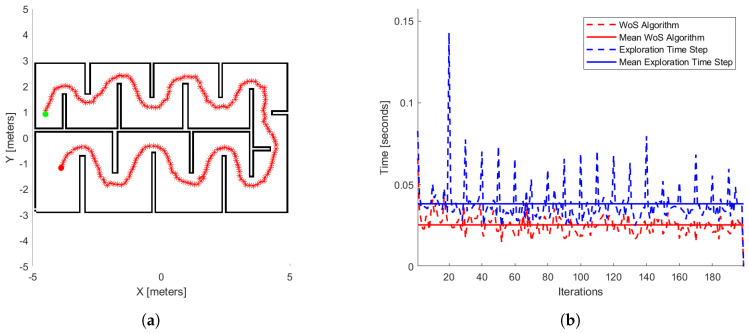
The workspace for comparative study (**a**). Green and red dots are the start and end agent’s position, respectively. And red continues across the curve of the agent’s trajectory. The numerical results of this exploration process are also depicted (**b**). The red dashed line represents the process time per iteration of the WoS algorithm, and the blue dashed line shows the exploration method’s time per iteration. The solid red line indicates the mean processing time of the WoS algorithm, while the solid blue line represents the mean processing time of the exploration method.

Finally, in the last stage of our results, we run our exploration method 100 times for different initial positions in the workspace illustrated in Figure 17. The same process appears in the papers [22,25,38]. In Table 4, we have the time exploration process of each method and also ours. In Figure 17a, we see the exploration process of the mobile agent at a specific initial position, such as in [25] and in Figure 17b, where it presents the process time per iteration of the WoS algorithm (red dash line) and our exploration method (blue dash line). In Figure 17b, we have the mean processing time of the WoS algorithm, which is 0.0255 s and the method 0.0388 s. The computer’s hardware that runs the third part of the simulations operates with Win11 Home, AMD Ryzen 7 7840HS processor, and RAM 16 GB.

## 7. Discussion

Figure 13f and Figure 14f, show the final results of the agent’s exploration in the first part of the simulations, while Figure 15f and Figure 16f present the results of the experiment with the AmigoBot unit. These plots illustrate the completeness of the workspace mapping and the no-collision trajectories provided by the proposed control architecture. Nevertheless, some irregularities can be observed in the agent’s trajectory, especially in the workspace with obstacles of the first part. This lack of smoothness is influenced by the complexity of the environment and the tuning of the exploration parameters, particularly the boundary values and source parameters of the Poisson equation.

In Table 2, we introduce the metrics of the overall results and average execution time of the sub-routines of the first study. Notice that the exploration parameters are identical for both simulations (see Table 1). As we observe, the agent needs more iterations and processing time to explore the workspace with obstacles compared to the labyrinth. This is due to the higher complexity involved in the obstacle-cluttered workspace and the fact that the exploration area remains large over many iterations, which is attributed to a result of the weakness of the Dead-End algorithm to manage such workspaces. This behavior is also reflected in the average time of the *Dead-End* sub-routine, which increases as the number of iterations grows, since more regions have to be checked. Thus, the time of the iteration process and particularly of the WoS algorithm increases in proportion to the complexity and the area of the explored region.

Similarly, in Table 3, we show the metrics of the second study, which involved real-world experiments using the AmigoBot. First of all, one notable observation is the relatively high average time of the *Extract Data* sub-routine. This is primarily due to the communication delay in transferring data from the AmigoBot to the PC during the experiment as well as the overhead introduced by the dead-end removal process, which is affected by the low map resolution (see Table 1). However, notice that the agent needs more iterations in the first workspace (labyrinth) than the second one (workspace with obstacles). This happens because the labyrinth involves narrow corridors; as a result, the agent is close enough to the workspace boundary and thus reduces its velocity. Furthermore, since the agent starts near the center, it must explore three separate sub-regions of the labyrinth. This behavior is evident in the high average time of the *Agent’s Movement* and *Iteration Process* sub-routines. However, the average time of the *WoS Algorithm* and *Iteration Process (Without Movement)* are less than the obstacle-cluttered workspace, since the Dead-End algorithm decreases significantly the explored region area.

Regarding the comparative simulation results, Table 4 presents the numerical results for the mean exploration time per iteration. According to these results, our method overcomes the methods presented by [22,38] in this metric. Our performance is also marginally better than [25], with a difference of only 0.028 s. Furthermore, comparing our Figure 17a with the corresponding figure in [25], we observe a significant distinction. While the author’s method exhibits a better exploration time, its solution time increases as the area of exploration space increases. In contrast, our method maintains an exploration time that remains close to its average value, due to applying the Dead-End algorithm—that reduces the exploration space—and the behavior of the WoS algorithm.

## 8. Conclusions

In this work, we proposed a novel method for autonomous exploration in unknown 2D indoor environments with obstacles using harmonic potential fields and the Monte Carlo Integration method. Velocity commands are computed by solving a Poisson equation with Dirichlet boundary conditions, placing a source point at the agent’s closest free frontier. The adapting WoS method ensures efficient, grid-free computation and direct gradient estimation. Moreover, the use of HVG plays a vital role in the exploration process, as the agent may possibly become stuck in the flat gradient region without it. Finally, real-world validation on an AmigoBot with LiDAR verified smooth, deadlock-free navigation in cluttered workspaces, and also a comparison study highlighted the advantages of our method. For future work, we aim at applying the exploration process to a CrazyFlie [39] equipped with a range sensor and expanding the method to 3D environments like a block of flats.

## Figures and Tables

**Table 1:** Values of exploration method parameters for two parts.

Exploration Parameter Values		Part A: Matlab		Part B: Real-World		Part C: Comparison
		Simulation 1 Labyrinth	Simulation 2 Workspace with Obstacles	Simulation 1 Labyrinth	Simulation 2 Workspace with Obstacles	
Agent						
	$p_{0}$	[4.0 m, 1.0 m, 0.0rad]	[−4.25 m, −4.25 m, 0.0 rad]	[0 m, 0 m, 0 rad]	[0 m, 0 m, 0 rad]	[−4.50 m, 0.92 m, 0 rad]
	ρ	0.3 m	0.3 m	0.3 m	0.3 m	0 m
Range Sensor						
	$R_{max}$	4 m	4 m	3 m	3 m	2 m
Map						
	$m_{r}$	0.0833 m/cell	0.0833 m/cell	0.05 m/cell	0.05 m/cell	0.0667 m/cell
WoS						
	$N_{G}$	10	10	8	8	8
	*N*	100	100	100	100	100
	ε	0.667 m	0.667 m	0.05 m	0.05 m	0.0533 m
Control Law						
	$K_{l}$	0.25	0.25	0.18	0.2	0.15
	$K_{a}$	0.25	0.25	0.25	0.2	0.2
	α	0.3 m	0.3 m	0.3 m	0.3 m	0.05 m
Boundary and Source Condition						
	$k_{f}$	5	5	2.5	5	5
	$k_{o}$	0.5	0.5	0.05	0.5	0.2
	$k_{s}$	40	40	16	20	50
Dead-End						
	$N_{D}$	1	1	10	10	10

**Table 2:** Simulation results of part A: Matlab.

	Simulation 1: Labyrinth	Simulation 2: Workspace with Obstacles
**Time Average**		
Extract Data	0.0683 s	0.0109 s
WoS Algorithm	0.0859 s	0.1419 s
Dead-End	0.0152 s	0.1134 s
Iteration Process	0.1695 s	0.2662 s
**Total Results**		
Iterations	149	180
Trajectory Length	36.97 m	44.34 m
Exploration Time	110.24 s	155.20 s

**Table 3:** Simulation results of part B: real world.

	Simulation 1: Labyrinth	Simulation 2: Workspace with Obstacles
**Time Average**		
Extract Data	0.4880 s	0.4976 s
WoS Algorithm	0.1373 s	0.1967 s
Agent’s Movement	4.0861 s	2.9283 s
Dead-End	0.1644 s	0.2196 s
Iteration Process (Without Movement)	0.7897 s	0.9139 s
Iteration Process	4.8758 s	3.8421 s
**Total Results**		
Iterations	82	47
Trajectory Length	13.72 m	7.52 m
Exploration Time	399.80 s	180.58 s

**Table 4:** Numerical results of part C: comparison study.

Methods	[25]	[22]	[38]	Ours
Mean Exploration Time Process	0.065 s	1.210 s	3.710 s	**0.037** s

## Data Availability

The original contributions presented in this study are included in the article. Further inquiries can be directed to the corresponding authors.

## Abbreviations

The following abbreviations are used in this manuscript:

A-SLAM	Active Simultaneous Localization and Mapping
BVP	Boundary Value Problem
FMBEM	Fast Multipole accelerated Boundary Element Method
HPF	Harmonic Potential Field
HVG	Hybrid Visibility Graph
MCI	Monte Carlo Integration
NBV	Next Best of View
PDE	Partial Differential Equation
RL	Reinforcement Learning
ROS	Robot Operating System
RRT	Rapidly Exploring Random Tree
SLAM	Simultaneous Localization and Mapping
WoS	Walking on Sphere

## Appendix A. Monte Carlo Integration

According to [27], Monte Carlo Integration is a stochastic numerical method for estimating the value of integrals in a specific domain. Hence, let function f(x):A→R be an $L^{1}$ integrable function on a domain A. Then, the integral $I:=\int_{A}f(x)dx$ is equal to the expected value of the Monte Carlo estimator:

(A1) $$F_{N}=\frac{\left|A\right|}{N}\sum_{i=1}^{N}f(X_{i}),X_{i}∼U\left(A\right),N>0$$

The error of $F_{N}$ is characterized by the variance, e.g., the squared deviation from the true value of *I*. In some cases, when we sample arbitrary points uniformly, there is a possibility of selecting points from regions that have little influence on the value of the integral *I*. As a result, the variance of the estimator may increase. To address this, we use the general Monte Carlo estimator with importance sampling, as presented below.

(A2) $$F_{N}=\frac{1}{N}\sum_{i=1}^{N}\frac{f(X_{i})}{p^{A}\left(X_{i}\right)},X_{i}∼p^{A},N>0$$

This approach focuses sampling on more significant regions and helps reduce the variance of the integral *I*. Moreover, an important advantage of the Monte Carlo estimator is that the number of samples *N* can be chosen arbitrarily, regardless of the dimension of the integrand. With this property, the Monte Carlo estimator is more efficient for calculating integrals in any n-dimensional space over traditional deterministic quadrature techniques.

## Appendix B. Harmonic Green Function in 2D

The free-space Green’s function in 2D for two points *x* and *y* is given by:

(A3) $$G^{R^{2}}\left(x,y\right)=\frac{1}{2\pi }ln\left(r\right)$$

where r:=||y−x||.

For a ball B(x,R) of radius *R* centered at *x*, we can derive

(A4) $$G^{B(x,R)}\left(x,y\right)=\frac{1}{2\pi }\ln\left(\frac{R}{r}\right)$$

These functions integrate over the ball B(x,R) to:

(A5) $$\left|G^{B(x,R)}\left(x\right)\right|:=\int_{B(x,R)}G^{B(x,R)}\left(x,y\right)dy=\frac{R^{2}}{4}$$

And the gradient w.r.t. *x* is given by:

(A6) $$\nabla G^{B(x,R)}\left(x,y\right)=\frac{y-x}{2\pi }\left(\frac{1}{r^{2}}-\frac{1}{R^{2}}\right)$$
